# A Novel CT to Cone-Beam CT Registration Method Enables Immediate Real-Time Intraprocedural Three-Dimensional Assessment of Ablative Treatments of Liver Malignancies

**DOI:** 10.1007/s00270-018-1909-0

**Published:** 2018-02-28

**Authors:** Marco Solbiati, Katia M. Passera, S. Nahum Goldberg, Alessandro Rotilio, Tiziana Ierace, Vittorio Pedicini, Dario Poretti, Luigi Solbiati

**Affiliations:** 1 R&D Unit, R.A.W. Srl, Busto Arsizio, VA Italy; 2 Department of Radiology, Hadassah Hebrew University Medical Centre, Jerusalem, Israel; 3 Department of Radiology, Beth Israel Deaconess Medical Center, Boston, MA USA; 4 Department of Radiology, Humanitas Clinical and Research Center, Rozzano, Milan Italy; 5 Department of Biomedical Sciences, Humanitas University, Rozzano, Milan Italy

**Keywords:** Interventional radiology, Microwave, Ablation, Liver tumors, Cone-beam CT

## Abstract

**Aim:**

To evaluate a novel contrast-enhanced cone-beam computed tomography (CE-CBCT) registration method for accurate immediate assessment of ablation outcomes.

**Materials and Methods:**

Contrast-enhanced computed tomography (CECT) was registered with CE-CBCT by applying semiautomatic landmark registration followed by automatic affine and non-rigid registration to correct for respiratory phase differences and liver deformation. This scheme was retrospectively applied to 30 patients who underwent 38 percutaneous microwave liver ablations. Three datasets were obtained for each case: (1) conventional CECT scans 24 h before ablation, (2) intraprocedural CE-CBCT scans, and (3) CECT scans 24 h post-ablation. Using a five-point scale, two experienced radiologists qualitatively assessed registration quality, equivalence of CE-CBCT assessment of ablation outcome to 24 h post-ablation CECT, and perceived increase of confidence using the fusion method to CBCT alone. Additionally, residual post-ablation tumor volumes were measured at both CE-CBCT and 24 h CECT and compared to the pre-CECT.

**Results:**

Registration quality was high for both radiologists (R1: 4.3 ± 0.6, R2: 4.4 ± 0.5; *p* = 0.87). Comparisons between the registration of pre-ablation CECT with CE-CBCT versus post-ablation CECT regarding the position of the ablated area to the treated target (R1: 4.4 ± 0.6, R2: 4.6 ± 0.4) and treatment outcome (R1: 4.5 ± 0.5, R2: 4.6 ± 0.4) were equivalent (*p* > 0.35). Increased confidence was noted when using fusion (R1: 4.6 ± 0.4, R2: 4.6 ± 0.4; *p* = 0.84). Moreover, in 6 ablations (15.8%) the intraprocedural registered CBCT showed residual tumor precisely where identified on the 24 h post-ablation CECT.

**Conclusions:**

Combined CE-CBCT holds the potential to change the current workflow of mini-invasive cancer local treatments. Given earlier visual identification of residual tumor post-ablation, this includes potentially eliminating the need for some additional treatments.

## Introduction

Image-guided ablation using radiofrequency or microwaves (MWA) has gained widespread attention and broad clinical acceptance as minimally invasive treatment of liver malignancies, particularly in non-surgical candidates [1–4]. Their success, however, relies strongly upon the operator’s skill and experience. Indeed, good clinical outcomes are predicated upon accurate selection criteria (i.e., tumor size, number, location, distance from major blood vessels), precise placement of ablative device(s), selection of optimal ablation parameters, and a thorough and accurate assessment of the completeness of treatment post-ablation. Lack of local tumor progression can only be confidently inferred when the ablation zone extends 5–10 mm beyond the entire tumor [5–7]. Real-time assessment could enable immediate further treatment when this desired periablational margin is not achieved, potentially avoiding subsequent additional treatment sessions.

Currently, many liver tumor ablations are performed using ultrasonography, where despite combined use of contrast-enhanced sonography and fusion imaging, immediate assessment of ablative margins can be hampered by incomplete three-dimensional evaluation [8, 9]. Likewise, when using CT guidance, the ablation margin is most often assessed subjectively—comparing pre-ablation contrast-enhanced CT (CECT) or MRI with post-ablation CECT. This is often accomplished solely by manually measuring distances from the tumor edge or ablation zone to selected landmarks in both studies. This is time-consuming and does not allow precise, quantitative three-dimensional assessment of ablative margins, given the subjective spatial co-registration of pre-ablation tumor volume with post-ablation necrosis. In order to overcome such limitations, software for three-dimensional co-registration has recently become available, but has yet to make a widespread impact on this clinical need [10].

Nowadays, contrast-enhanced cone-beam CT (CE-CBCT) is increasingly used in local treatments of liver malignancies, including both transcatheter chemoembolization and percutaneous ablation. Despite poorer resolution, CE-CBCT provides fast generation of volumetric images with lower radiation dose compared to conventional CT, enabling the interventionalist to plan and directly guide procedures based upon immediate assessment of results achieved [11–15]. Yet, comparison of post-ablation CBCT with pre-ablation CECT scans is very cumbersome as CT and CBCT datasets differ with respect to many parameters, and software providing fast and accurate three-dimensional co-registration of CECT and CE-CBCT is currently not available.

To surmount this issue, we introduce here a novel method that combines state-of-the-art image processing algorithms to provide precise and fast spatial co-registration of pre-treatment CECT with immediate post-treatment CE-CBCT, to enable accurate assessment of ablative margins in three dimensions immediately post-ablation.

## Materials and Methods

### Pre-ablation Registration of CECT with CE-CBCT

Registration of CECT with CE-CBCT consists of four steps: (1) pre-ablation CECT re-sampling at CBCT resolution, (2) semiautomatic rigid registration, (3) automatic affine registration followed by nonlinear registration, and (4) overlapping of pre-ablation CECT segmented over the registered target on CE-CBCT.

The semiautomatic rigid registration is based upon selection of three corresponding landmarks visible in both image datasets. Here, intrahepatic blood vessels were selected as these are closer to the target and have less risk of deformity from respiratory motion than the outer contour of the liver [16]. This step is necessary as CBCT and CT images often do not completely overlap and automatic registration cannot correct this large mismatch. To correct for different liver shapes, different breathing phases and motion artifacts, subsequent affine and nonlinear registrations are automatically performed. For this procedure, CE-CBCT is considered the fixed (i.e., template) imaging modality with CECT the moving (i.e., superimposed) image. To accomplish this, the CECT is warped upon the CE-CBCT in order to achieve registration. The method is implemented using Insight Toolkit (ITK) libraries and Elastix toolbox [17, 18]. Normalized mutual information was used for registration as it offers the best performance in cases of multimodality. Nonlinear registration is based on B splines [17].

### Protocol

Our CECT-CE-CBCT co-registration algorithm was retrospectively applied to 30 patients (22 males and 8 females, aged 65–85 years) who underwent 38 percutaneous microwave liver ablations (MWA) (28 hepatocellular carcinomas (HCCs) in 22 patients and 10 cases of colorectal metastases (MET) in 8 patients). Tumor diameters ranged from 0.7 to 3.3 cm (0.38–14.49 cc volume) (Table 1). One patient underwent two treatments, since in the first session one tumor was not fully ablated.

**Table 1:** Patients and treatment data

Treatment	Patient (male/female, age)	Tumor type	Tumor location (segment)	Tumor volume (cm^3^)	Post-CT ablation volume (cm^3^)	Residual tumor volume percentage (cm^3^ %)
1	M, 71	HCC	6	0.8	19.1	0
2	M, 81	HCC	8	2.1	47.1	0.03 (1.4%)
		*HCC*	*2*	*0.5*	*5.0*	*0.5 (100%)*
3	M, 70	HCC	4	3.2	43.7	0.2 (6.3%)
4	M, 81^a^	HCC	2	0.6	28.2	0.01 (1.2%)
5	F, 74	MET	7	11.2	39.2	0.2 (5.8%)
6	M, 75	HCC	2	13.6	18.2	0.6 (14.3%)
7	F, 80	HCC	4	0.9	3.0	0
8	M, 85	**HCC**	**5**	**11.7**	**25.0**	**3.3 (28.3%)**
9	M, 70	HCC	8	4.8	10.5	0
10	M, 71	HCC	5	1.1	5.3	0
11	M, 76	MET	8	0.5	2.9	0.01 (12.8%)
12	M, 82	HCC	6	0.5	3.3	0
13	M, 71	MET	4	1.0	5.2	0
14	M, 80	HCC	4	11.1	35.7	1.3 (11.5%)
15	F, 65	**MET**	**2**	**1.0**	**15.4**	**0.6 (58.1%)**
16	M, 79	HCC	8	1.5	9.7	0
		HCC	2	2.5	9.3	0
17	M, 77	HCC	3	9.4	23.9	0
18	M, 70	HCC	5	1.4	2.9	0
		HCC	4	5.0	10.0	0
19	F, 83	HCC	8	2.8	4.0	0
20	F, 76	HCC	8	0.6	3.7	0
		HCC	4	0.8	5.3	0
21	M, 81	HCC	5	4.7	12.0	0
22	F, 72	HCC	6	5.7	7.6	0.6 (10.2%)
23	M, 81	**HCC**	**2**	**6.9**	**13.0**	**1.7 (24.2%)**
24	M, 72	HCC	4	14.5	20.9	0
		*HCC*	*3*	*0.8*	*4.0*	*0.8 (100%)*
25	M, 82	MET	2	10.3	22.9	0
26	M, 81	HCC	2	0.4	18.8	0
27	F, 75	HCC	5	2.5	6.6	0
		**HCC**	**2**	**1.2**	**7.0**	**0.4 (31.7%)**
28	M, 78	MET	7	0.8	3.8	0
		MET	8	0.4	2.9	0
29	F, 78	MET	8	2.5	4.0	0
		MET	2	1.1	5.3	0
30	M, 77	MET	2	0.6	3.0	0

Residual tumor volume means the volume of the portion of unablated tumor as a result of incomplete ablation

The totally missed targets (italics) and the targets with a volume of unablated tumor ranging from 20 to 58.1% of the initial volume (bold) are highlighted

^a^Treatment 2 and Treatment 4 were performed in the same patient

For each case, three datasets were obtained: (1) conventional CECT scans 24 h before ablation, (2) intraprocedural CE-CBCT scans, and (3) CECT scans 24 h after ablation.

Pre- and post-ablation CT examinations were performed using a 64-slice scanner (GE Healthcare, Milwaukee, USA) after intravenous administration of 120–140 cc (mean, 129 ± 8) non-ionic contrast medium (iomeprol, Iomeron 300; Bracco, Milan, Italy) at 3 cc/s. Automatic bolus tracking (20 ml at 3 cc/s) was used, with monitoring scans acquired starting after a delay of 8 s. Approximately 6–9 s thereafter, the contrast enhancement threshold (90 HU) was reached within the ROI (i.e., the lumen of the descending aorta), and arterial phase scans were acquired. Portal venous phase scans automatically started 15 s after completion of the arterial phase scans. Both phases were performed with 3–5 mm collimation and 2–2.5 mm reconstruction intervals, with a matrix of 512 × 512 pixels, and in-plane pixel size of 0.48–0.86 mm (mean 0.68 ± 0.07).

Five to ten minutes following cessation of microwave ablation, post-ablation CE-CBCT dataset volumes including the entire patients’ livers were acquired at CBCT (Artis zee, Siemens Healthcare, Erlangen, Germany) after intravenous administration of 120 cc of iomeprol at 3 cc/s. Arterial and portal venous phase axial scans were triggered 3 and 30 s after the injection of contrast medium and reconstructed perpendicular to the patient’s longitudinal axis. Scanning parameters were 1.2 m source detector distance, 1.5° rotation step, 5 s rotation duration, 200° total arc trajectory range, 128 images (projections), 0.36 microGy/frame radiation dose, and 2–5 mm reconstruction intervals. Each reconstructed CT slice had a 512 × 512 pixels matrix, with an in-plane pixel size of 1.02–1.20 mm (mean 1.18 ± 0.3). Each 3D scan covered an approximate volume of 250 × 200 × 200 mm.

### CT Image Preprocessing

For registration, the arterial phase scans were used as they provided the greatest enhancement of the tumors.

Pre-ablation and post-ablation CECT images were filtered with a diffusion filter in order to reduce noise [19] for automatic segmentation of liver, tumor, and resultant ablation zone. For liver segmentation, a fast marching and 3D geodesic active contour program identified and selected the entire liver [20]. As a final preprocessing step that finalized the CECT segmentation, tumor and induced coagulation necrosis were further segmented using a hybrid of fuzzy c-means algorithm and random walkers method based upon CT density [21].

### Clinical Evaluation

Two radiologists, each with a history of more than 2000 ablation procedures, performed, evaluated our method, based upon three criteria: registration quality, correspondence of the assessments of ablation outcomes and perceived increase of confidence for treatment evaluation.

Registration quality was assessed by visually comparing images before and after the overlapping registration of pre-ablation CECT images over CE-CBCT images. Next, the radiologists evaluated the accuracy of post-ablation outcomes comparing pre-ablation CECT fused to intraprocedural immediate CE-CBCT with that of 24 h post-ablation CECT. Both registration quality and ablation outcomes assessments were performed according to a qualitative five-point scale (1: poor—2: fair—3: satisfactory—4: good—5: excellent). Spatial position accuracy was defined as displacement between the tumor location on the pre-ablation CT when overlapped to CBCT and post-ablation CT (1: > 7 mm, 2: 5–7 mm, 3: 3–5 mm, 4: 1–3 mm, 5: < 1 mm).

Finally, evaluation of the radiologist’s perceived improved confidence in achieving complete ablation over simple visualization of the CBCT image set without image registration was performed using a 1–5-scale scoring system (1: no confidence increase, 2: low 3: discrete/moderate 4: high, and 5: very high). The results of all four gradings by the radiologists were subject to inter-observer analysis, including direct comparison using Student’s *T* test.

## Results

**Fig. 1:**
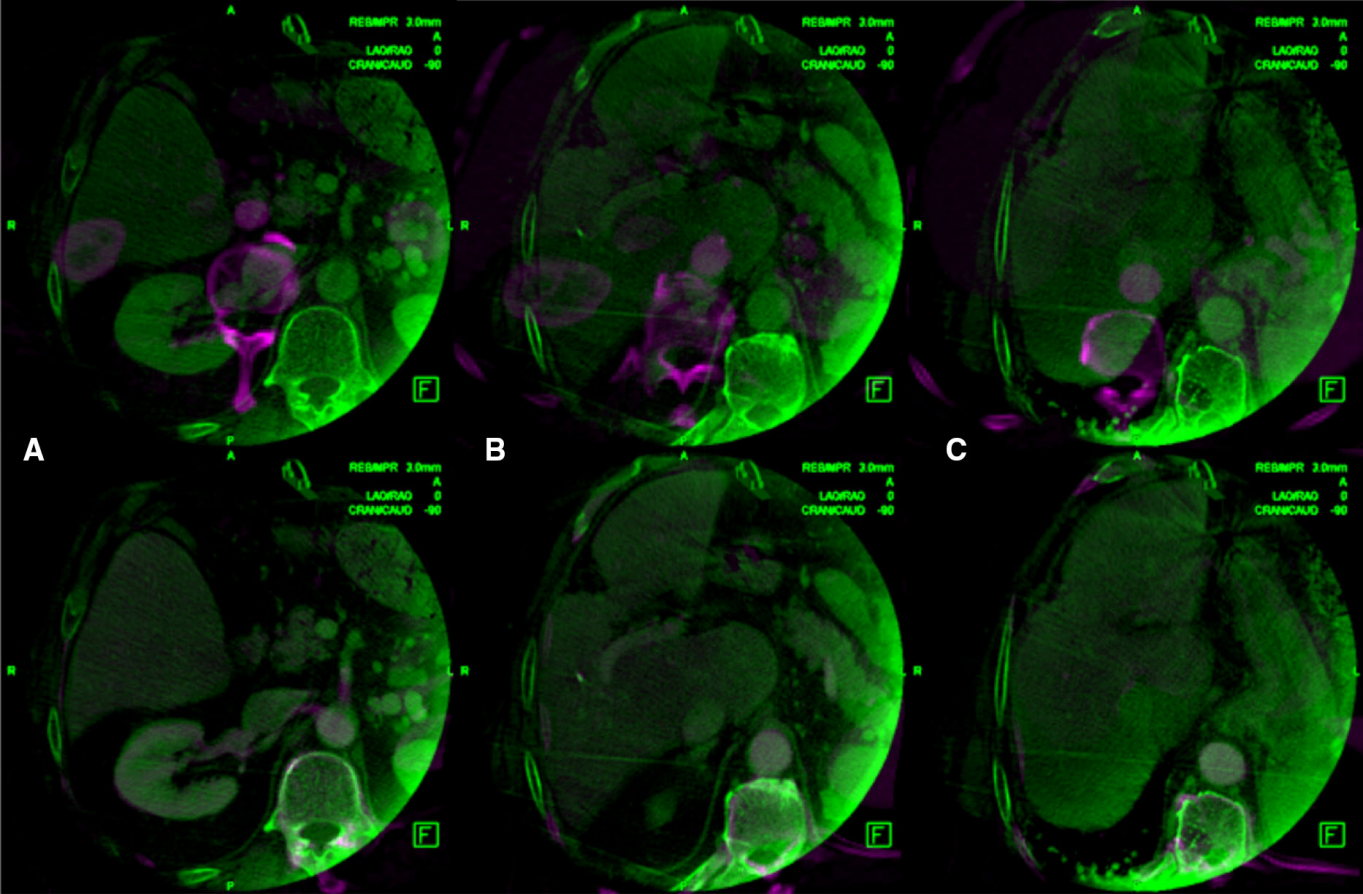
Effects of image registration. Unregistered pre-CT (in purple color) overlapped to CBCT (in green color) (first row) compared to registered pre-CT overlapped to CBCT (second row) for three representative slices of patient #1. The comparison shows that the substantial misalignment occurring before registration (manifest as a purple shadow) was almost completely eliminated using our new fusion method despite the technical differences between the two different imaging modalities

Whole liver imaging was possible in all patients, based upon accurate pre-procedural patient centering. The average time required to perform the entire procedure of landmark selection and co-registration ranged from 30 to 120 s. An example of the registration achieved is shown in Fig. 1.

The quantitative results of the clinical evaluation are summarized in Table 2. Overall, extremely high concordance was noted between both radiologists with identical grades of 43.3, 37.4, 78.9, and 60.5% assigned for registration quality, position, clinical indication, and confidence improvement, respectively, and with no case noted of discordance by more than one category (Table 2).

**Table 2:** Results of clinical evaluation

Treatment	Registration quality		Tumor	Target position (segment)	Correspondence with post-CT				Confidence improvement	
					Position		Clinical indication			
	R1	R2			R1	R2	R1	R2	R1	R2
1	5	4	HCC	6	5	5	5	5	5	5
2	3	3	HCC	2	3	3	3	3	4	5
			HCC	8	4	3	4	4	4	5
3	4	4	HCC	4	4	5	5	5	5	4
4	3	4	HCC	2	4	5	5	5	5	5
5	5	5	MET	7	4	5	4	5	5	5
6	5	5	HCC	2	5	5	5	5	5	5
7	4	5	HCC	4	5	4	5	5	5	5
8	5	4	HCC	5	4	5	4	5	5	4
9	5	5	HCC	8	5	5	5	5	5	5
10	4	5	HCC	5	5	4	5	5	5	5
11	5	4	MET	8	5	4	5	5	5	5
12	5	4	HCC	6	5	5	4	4	4	5
13	4	5	MET	4	4	5	5	5	5	4
14	2	3	HCC	4	4	4	4	4	4	3
15	3	3	MET	2	3	4	4	3	4	4
16	5	4	HCC	8	4	5	4	4	4	4
			HCC	2	3	4	3	3	3	4
17	5	5	HCC	3	4	5	4	4	4	5
18	5	5	HCC	5	5	5	5	5	5	5
			HCC	4	5	5	5	5	5	5
19	4	5	HCC	8	4	5	5	4	5	5
20	5	5	HCC	8	5	5	5	5	5	5
			HCC	4	5	5	5	5	5	5
21	5	4	HCC	5	4	4	3	3	3	4
22	4	4	HCC	6	5	4	5	5	5	4
23	5	4	HCC	2	4	5	4	5	4	4
24	4	5	HCC	4	5	5	5	5	5	4
			HCC	3	5	4	5	5	5	5
25	5	4	MET	2	5	5	5	5	5	4
26	3	4	HCC	2	5	4	5	4	5	5
27	5	5	HCC	5	4	5	4	5	4	5
			HCC	2	4	5	4	5	4	5
28	5	5	MET	7	4	4	4	4	4	4
			MET	8	5	4	5	5	5	5
29	5	5	MET	8	5	5	5	5	5	5
			MET	2	5	5	5	5	5	5
30	4	5	MET	2	5	5	5	5	5	5
Average	4.3	4.4			4.4	4.6	4.5	4.6	4.6	4.6
Standard deviation	0.6	0.5			0.6	0.4	0.5	0.4	0.4	0.4

Registration quality was on average scored extremely high for both radiologists (R1: 4.3 ± 0.6, R2: 4.4 ± 0.5, mean ± standard deviation; *p* = 0.87), with no grade 1 or 2 observations recorded. Comparisons between the registration of pre-ablation CECT with CE-CBCT to pre-ablation CECT with post-ablation CECT for both position of the ablated area with the treated target (R1: 4.4 ± 0.6, R2: 4.6 ± 0.4) and treatment outcome (R1: 4.5 ± 0.5, R2: 4.6 ± 0.4) were equivalent (*p* = 0.36 and 0.73, respectively). Finally, a demonstrably higher increase of confidence was noted for both radiologists (R1: 4.6 ± 0.4, R2: 4.6 ± 0.4; *p* = 0.84) when they could replace the simple visual inspection of CBCT images without any registration with the proposed method of spatial overlapping of CBCT images with pre-ablation images. A full 75/78 of comparisons (96.1%) were rated as “high” or “very high” with no grade 1 or 2 observations were noted by either radiologist and only 3 of 78 observations (3.9%) rendered grade 3 as “discrete/moderate.”

**Fig. 2:**
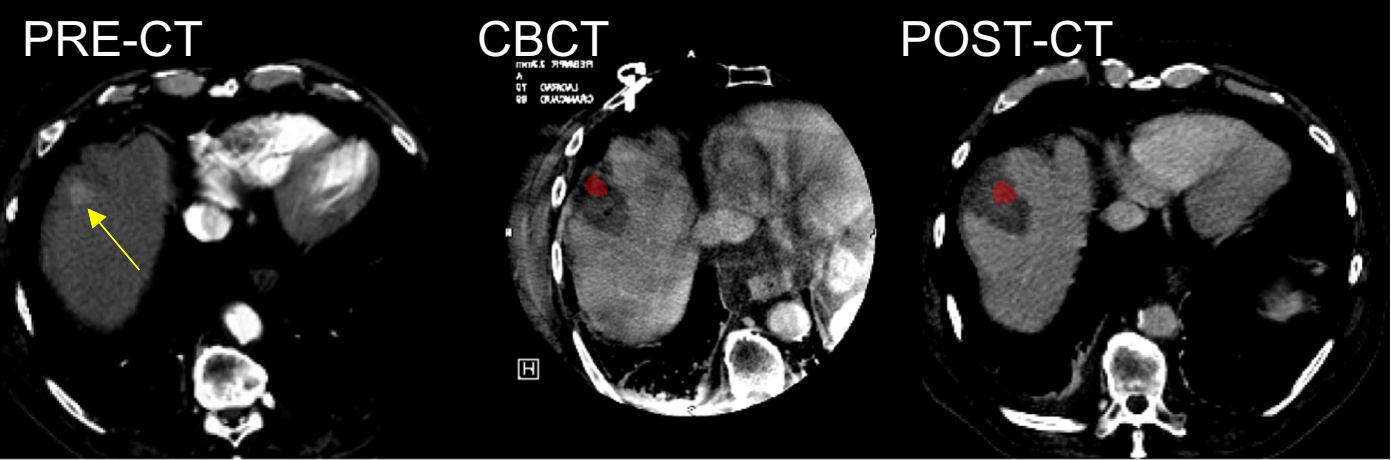
Demonstration of successful ablation treatment at CBCT. First HCC of patient #2, studied with CECT before ablation (PRE-CT) (HCC indicated by yellow arrow), intraprocedural CBCT co-registered with PRE-CT (with the pre-treatment HCC colored in red) and 24-h post-ablation with CECT (post-CT) (with the pre-treatment HCC colored in red). Both CBCT and post-CT show the ablated area entirely surrounding the target

**Fig. 3:**
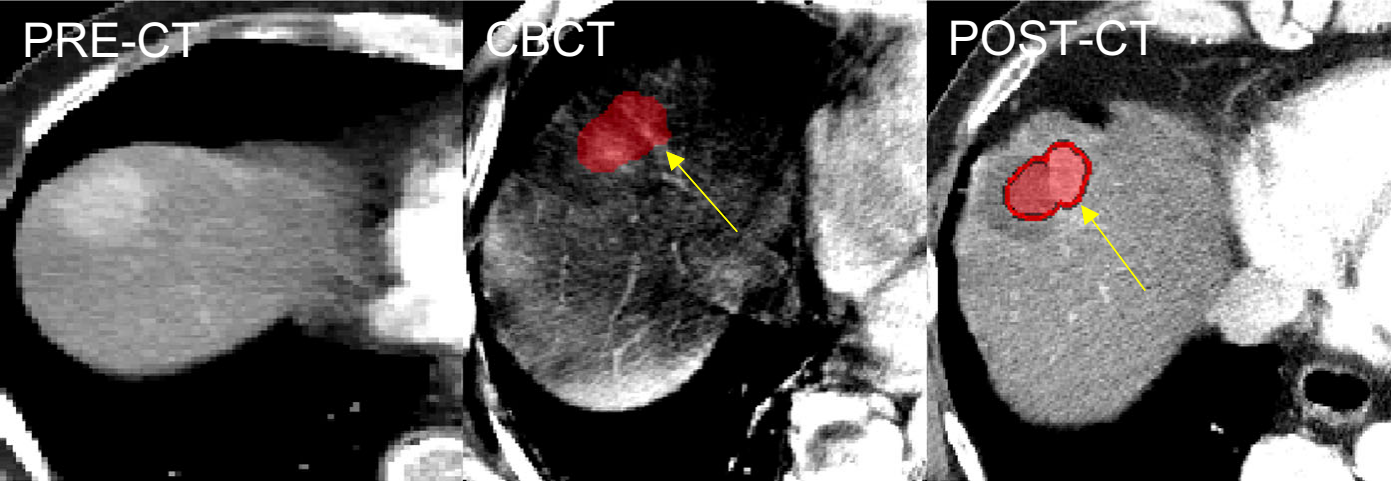
Incomplete treatment detected by CBCT. Intraprocedural CBCT registered with PRE-CT in patient #8 of Table 1 shows partial failure of the ablation treatment, as confirmed by the 24 h post-CT. The medial portion (yellow arrows) of the HCC (colored in red) is not entirely covered by the ablated area both on CBCT and on post-CT

**Fig. 4:**
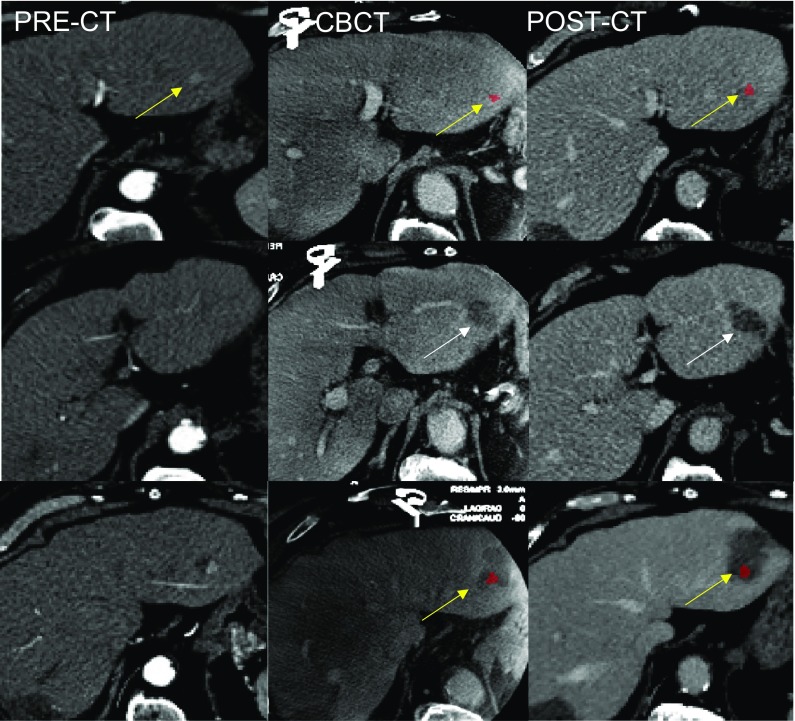
CBCT demonstration of missed target. Comparison among pre-ablation CT (PRE-CT), intraprocedural registered CBCT, and 24 h post-ablation CT (post-CT) (second HCC of patient #2). After the first treatment, both CBCT and post-CT show that the HCC (colored in red and indicated by yellow arrows) is entirely located outside the volume of ablation (missed target) (top row). Most notably, the ablated area (indicated by the white arrows of the second row) is located caudally with respect to the actual target position (yellow arrows of the first row) both on CBCT and post-ablation CT. In the bottom row, the successful retreatment is shown, with the ablated area (yellow arrows) fully covering the target (red dot)

Moreover, with registration of pre-CECT with CE-CBCT the amount of residual post-ablation tumor volume was measured. In 24/38 (63.2%) ablations, the registration did not show any residual tumor and for all 24/24 (100%) of them no residual tumor was also detected on the 24 h post-ablation CECT (Fig. 2). In 12/38 (31.6%) ablations, the intraprocedural registered CBCT showed residual unablated tumor, ranging from 1.2 to 58.1% (mean 17.2 ± 16%) of the initial tumor volume. In 4/12 (33.3%) cases, the amount of unablated tumor ranged from 20 to 58.1% of the initial tumor volume and for all 4 of these the 24 h post-ablation CECT showed a rim of residual enhancement indicating incomplete ablation [22] (Fig. 3). In the remaining 8/12 (66.7%) cases, the amount of unablated tumor detected by CBCT ranged from 1.2 to 14.3% of the initial volume. In these 8 (100%), the 24 h post-ablation CECT did not detect any residual enhancement. In the last 2/38 (5.3%) treatments, both intraprocedural registered CBCT and 24 h post-ablation CECT showed that the target was completely missed (Fig. 4).

Thus, ultimately in 6/38 (15.8%) cases (4/38 (10.5%) with the amount of unablated tumor ranging from 20 to 58.1% and 2/38 (5.3%) with a completely missed target) our CBCT fusion method identified the need for retreatment as confirmed by the post-ablation CECT.

## Discussion

We describe a novel registration method between post-ablation CBCT and pre-ablation CECT scans that facilitates intraprocedural treatment assessment.

Although CT and CBCT are both based upon radiation and computed tomography, their datasets differ with respect to many parameters including: the field-of-view focusing of interest, speed of rotation, susceptibility to motion, different signal-to-noise ratios, image resolution and CBCT scan artifacts that may lead to non-uniform intensity images [23]. Additionally, liver parenchyma often undergoes significant deformation (particularly in cranial segments) due to respiratory movement [24]. These issues make co-registration of the two volume datasets non-intuitive and challenging.

Prior studies addressing CBCT registration in different anatomic sites [25, 26], including liver [23, 27–29] have predominantly focused upon adaptive radiation therapy. Image fusion of pre-treatment CT with post-treatment CBCT for the assessment of ablation margins has been previously described, but has been performed through landmark rigid registration, which does not take into account modifications of liver and target tumor shape induced by patient breathing and/or movement—factors shown to impede ablation success [24, 30, 31]. However, our algorithm surmounts these issues by providing automatic rigid and non-rigid registration.

Equivalent quality and confidence were noted for both types of fusion images by the two radiologists. Furthermore, despite substantial clinical experience, confidence in their assessment of the results obtained was increased over that achieved by only visually inspecting CBCT images. Particularly, in all the ablations (6/38, 15.8%) where the intraprocedural registered CBCT showed residual tumor percentage greater than 15%, on the 24 h post-ablation CECT the two radiologists visually identified residual tumor enhancement exactly where the CBCT predicted it, leading to the conclusion that if the proposed method had been employed, and in these six tumors the second treatment could have been avoided. In the 8/38 (21.1%) treatments in which CBCT showed minimal (less than 14.3%) amount of residual tumor volume, the 24 h post-ablation CECT did not detect any residual enhancement likely due to the very small amount of residual tumor and/or the presence of the peripheral rim of inflammation. Thus, studies including 3-month, 6-month, and 1-year follow-up dataset are needed to definitely confirm or exclude the presence of small areas of unablated tumor and determine the true sensitivity and specificity of each method. Likewise, although 15% residual enhancement proved to be a reasonable discriminator in this series, much more comprehensive study will be required to better define the precise residual tumor volume cutoff for retreatment, thus better characterizing the actual efficacy of this method.

Additionally, the method is suitable for real-time treatment assessment as it is very fast. The selection of the three landmarks in the two datasets requires only minimal user interaction and takes less than 2 min, while the rest of the registration is fully automatic and takes only a few seconds. After registration, overlapping the target tumor identified by conventional pre-ablation CECT (automatically segmented and registered) with CBCT images enables easier identification of untreated areas during the ablation.

In conclusion, this method potentially decreases patient risk and discomfort associated with the difficulties frequently encountered in the retreatment of the partially ablated tumors. Accordingly, the proposed method holds the potential to change the current workflow of interventional oncologic treatments. Future work will include both greater clinical evaluation and determination as to the utility of extending this approach to fusion of CBCT with other imaging modalities such as MRI or PET.

## References

[1] Ahmed M, Brace CL, Lee FT, Goldberg SN (2011). Principles of and advances in percutaneous ablation. Radiology.

[2] Gillams A, Goldberg SN, Ahmed M, Bale R, Breen D, Callstrom M (2015). Thermal ablation of colorectal liver metastases: a position paper by an international panel of ablation experts. The interventional oncology Sans Frontières Meeting 2013. Eur Radiol.

[3] Kim YS, Lim HK, Rhim H, Lee MW, Choi D, Lee WJ (2013). Ten-year outcomes of percutaneous radiofrequency ablation as first-line therapy of early hepatocellular carcinoma: analysis of prognostic factors. J Hepatol.

[4] Solbiati L, Ahmed M, Cova L, Ierace T, Brioschi M, Goldberg SN (2012). Small liver colorectal metastases treated with percutaneous radiofrequency ablation: local response rate and long-term survival with up to 10-year follow-up. Radiology.

[5] Hocquelet A, Trillaud H, Frulio N, Papadopoulos P, Balageas P, Salut C (2016). Three-dimensional measurement of hepatocellular carcinoma ablation zones and margins for predicting local tumor progression. J Vasc Interv Radiol.

[6] Wang X, Sofocleous CT, Erinjeri JP, Petre EN, Gonen M, Do KG (2013). Margin size is an independent predictor of local tumor progression after ablation of colon cancer liver metastases. Cardiovasc Interv Radiol.

[7] Kim YS, Lee WJ, Rhim H, Lim HK, Choi D, Lee JY (2010). The minimal ablative margin of radiofrequency ablation of hepatocellular carcinoma (> 2 and < 5 cm) needed to prevent local tumor progression: 3D quantitative assessment using CT image fusion. AJR Am J Roentgenol.

[8] Li K, Su Z, Xu E, Huang Q, Zeng Q, Zheng R (2017). Evaluation of the ablation margin of hepatocellular carcinoma using CEUS-CT/MR image fusion in a phantom model and in patients. BMC Cancer.

[9] Meloni MF, Andreano A, Zimbaro F, Lava M, Lazzaroni S, Sironi S (2012). Contrast enhanced ultrasound: roles in immediate post-procedural and 24-h evaluation of the effectiveness of thermal ablation of liver tumors. J Ultrasound.

[10] Luu HM, Klink C, Niessen W, van Walsum T (2015). An automatic registration method for pre- and post-interventional CT images for assessing treatment success in liver RFA treatment. Med Phys.

[11] Abi-Jaoudeh N, Venkatesan AM, Van der Sterren W, Radaelli A, Carelsen B, Wood BJ (2015). Clinical experience with cone-beam CT navigation for tumor ablation. J Vasc Interv Radiol.

[12] Bapst B, Lagadec M, Breguet R, Vilgrain V, Ronot M (2016). Cone beam computed tomography (CBCT) in the field of interventional oncology of the liver. Cardiovasc Interv Radiol.

[13] Floridi C, Radaelli A, Pesapane F, Fumarola EM, Lecchi M, Agostini A (2017). Clinical impact of cone beam computed tomography on iterative treatment planning during ultrasound-guided percutaneous ablation of liver malignancies. Med Oncol.

[14] Iwazawa J, Ohue S, Hashimoto N, Muramoto O, Mitani T (2012). Survival after C-arm CT-assisted chemoembolization of unresectable hepatocellular carcinoma. Eur J Radiol.

[15] Miyayama S, Yamashiro M, Hashimoto M, Hashimoto N, Ikuno M, Okumura K (2013). Identification of small hepatocellular carcinoma and tumor-feeding branches with cone-beam CT guidance technology during transcatheter arterial chemoembolization. J Vasc Interv Radiol.

[16] Dong Y, Wang WP, Mao F, Ji ZB, Huang BJ (2016). Application of imaging fusion combining contrast-enhanced ultrasound and magnetic resonance imaging in detection of hepatic cellular carcinomas undetectable by conventional ultrasound. J Gastroenterol Hepatol.

[17] Klein S, Staring M, Murphy K, Viergever MA, Pluim JP (2010). Elastix: a toolbox for intensity-based medical image registration. IEEE Trans Med Imaging.

[18] Shamonin DP, Bron EE, Lelieveldt BP, Smits M, Klein S, Staring M (2014). Fast parallel image registration on CPU and GPU for diagnostic classification of Alzheimer’s disease. Front Neuroinf.

[19] Lamecker H, Lange T, Seebass M. Segmentation of the liver using a 3D statistical shape model. ZIB-Report. 2004. p. 1–25.

[20] Luo S, Li X, Li J (2014). Review on the methods of automatic liver segmentation from abdominal images. J Comput Commun.

[21] Moghbel M, Mashohor S, Mahmud R, Saripan MI (2016). Automatic liver tumor segmentation on computed tomography for patient treatment planning and monitoring. EXCLI J.

[22] Ahmed M, Solbiati L, Brace CL, Breen DJ, Callstrom MR, Charboneau JW, Chen MH, Choi BI, de Baère T, Dodd GD, Dupuy DE, Gervais DA, Gianfelice D, Gillams AR, Lee FT, Leen E, Lencioni R, Littrup PJ, Livraghi T, Lu DS, McGahan JP, Meloni MF, Nikolic B, Pereira PL, Liang P, Rhim H, Rose SC, Salem R, Sofocleous CT, Solomon SB, Soulen MC, Tanaka M, Vogl TJ, Wood BJ, Goldberg SN, International Working Group on Image-Guided Tumor Ablation, Interventional Oncology Sans Frontières Expert Panel, Technology Assessment Committee of the Society of Interventional Radiology, Standard of Practice Committee of the Cardiovascular and Interventional Radiological Society of Europe (2014). Image-guided tumor ablation: standardization of terminology and reporting criteria—a 10-year update. J Vasc Interv Radiol.

[23] Zhang L, Chefd’hotel C, Ordy V, Zheng J, Deng X, Odry B. A knowledge-driven quasi-global registration of thoracic-abdominal CT and CBCT for image-guided interventions. In: SPIE Med Imaging. 2013. p. 867110–867111.

[24] Appelbaum L, Solbiati L, Sosna J, Nissenbaum Y, Greenbaum N, Goldberg SN (2013). Evaluation of an electromagnetic image-fusion navigation system for biopsy of small lesions: assessment of accuracy in an in vivo swine model. Acad Radiol.

[25] Cazoulat G, Simon A, Acosta O, Ospina J, Gnep K, Viard R, et al. Dose monitoring in prostate cancer radiotherapy using CBCT to CT constrained elastic image registration. In: Prostate Cancer Imaging. 2011. p. 70–79.

[26] Landry G, Nijhuis R, Dedes G, Handrack J, Thieke C, Janssens G (2015). Investigating CT to CBCT image registration for head and neck proton therapy as a tool for daily dose recalculation. Med Phys.

[27] Brock KK, Hawkins M, Eccles C, Moseley JL, Moseley DJ, Jaffray DA (2008). Improving image-guided target localization through deformable registration. Acta Oncol.

[28] Hawkins MA, Brock KK, Eccles C, Moseley D, Jaffray D, Dawson LA (2006). Assessment of residual error in liver position using kV cone-beam computed tomography for liver cancer high-precision radiation therapy. Int J Radiat Oncol Biol Phys.

[29] Zhen X, Gu X, Yan H, Zhou L, Jia X, Jiang SB (2012). CT to cone-beam CT deformable registration with simultaneous intensity correction. Phys Med Biol.

[30] Abdel-Rehim M, Ronot M, Sibert A, Vilgrain V (2015). Assessment of liver ablation using cone beam computed tomography. World J Gastroenterol.

[31] Iwazawa J, Hashimoto N, Mitani T, Ohue S (2012). Fusion of intravenous contrast-enhanced C-arm CT and pretreatment imaging for ablation margin assessment of liver tumors: a preliminary study. Indian J Radiol Imaging.

